# Optimizing prevention and community-based management of severe malnutrition in children

**DOI:** 10.1371/journal.pmed.1003924

**Published:** 2022-03-01

**Authors:** Zulfiqar A. Bhutta

**Affiliations:** 1 Institute for Global Health & Development, The Aga Khan University, South-central Asia, East Africa & United Kingdom; 2 Robert Harding Chair, Centre for Global Child Health, The Hospital for Sick Children, Toronto, Canada

## Abstract

Zulfiqar A. Bhutta discusses prevention and treatment strategies for optimization of community-based management of severe acute malnutrition in children.

In this issue of *PLOS Medicine*, Matt Hitchings and colleagues detail the findings from their prospective cluster-randomized crossover trial conducted across 10 health centers in Sokoto, Nigeria, to assess the nutritional recovery in children with uncomplicated severe acute malnutrition (SAM) receiving monthly follow-up compared to the standard weekly follow-up schedules [1]. In almost 4,000 children so allocated, the nutritional recovery at 3 months’ follow-up was lower in the monthly follow-up group (52.4%) compared to the standard weekly group (58.8%), with higher cumulative mortality at 3 months (8.5% versus 6.2% with the standard weekly follow-up). In contrast, rates of default and relapse were significantly lower among SAM children allocated to monthly follow-up. The authors, while urging caution in adopting a modified schedule of monthly follow-up visits in such children, also recognize the trade-off of simplicity and ease of operations in some settings where weekly follow-up visits are not feasible.

Despite global progress in improving maternal and child undernutrition, the high burden of severe malnutrition persists. Recent estimates show a small reduction (from 15.9% to 14.2%) in wasting prevalence in low-income countries, and a slight increase (from 3.3% to 4.7%) in middle-income countries, although overall almost 50 million children aged under 5 years still remain wasted worldwide [2]. This burden of SAM has most likely been exacerbated during the recent Coronavirus Disease 2019 (COVID-19) pandemic, with an estimated additional 6.7 million children becoming wasted in 2020 [3].

Within this large number of wasted children are those with SAM who are triaged to facility-based nutritional rehabilitation if seriously ill, or community-based treatment regimens if stable. The development of standardized management protocols for children with SAM with ready-to-use therapeutic foods (RUTFs) represents one of the greatest advances in treating such children at scale and reducing the mortality associated with the condition [4]. However, given the general context where childhood SAM clusters, such as those affected by extreme poverty, climate change, conflict, and involving displaced populations, major challenges remain in optimizing SAM management. These include relatively high rates of relapse [5], and associated residual mortality with severe malnutrition, often exceeding 10% in some settings [6]. Strategies are thus needed to optimize community case management aimed at simplifying the treatment regimen for SAM, reducing defaults and relapse rates among affected children.

Such real-life evaluations of management strategies for severe malnutrition among at-risk children are few and far between, and most welcome. The global evidence base for the management of SAM in various settings is still mixed, with wide variations in recovery or relapse rates and mortality. This is especially the case in complex emergencies and conflict settings [7] with obvious limitations of human resources and commodities. The challenges of managing SAM in different contexts and settings are directly related to available nutrition rehabilitation commodities and trained human resources, as well as the ability of poor and food-insecure households to follow complex regimen and follow-up schedules. For many poor households with daily wage laborers or workers, taking a day off to travel to ambulatory care settings is a weekly financial and logistic hardship that may be impossible to bear. Alternative approaches with community outreach workers providing care and commodities in domiciliary settings has also met with mixed success, with lower rates of uptake in effectiveness settings with busy public-sector workers [8,9].

There are additional research questions related to the nutritional rehabilitation and management of SAM including dosage schedules and protocols for administering RUTF in outreach and ambulatory programs. Additional therapeutic challenges in managing children with SAM include the limited repertoire of options for interventions in children under 6 months of age, as well as strategies to manage children with concurrent stunting and wasting [1,10]. While the recommendations for facility-based management of unstable children with SAM are well recognized [11], corresponding protocols for ambulatory management of severely malnourished children with suspected infections and at risk of adverse outcomes are still a subject of much debate [12].

The gains from potentially simplifying ambulatory management strategies for SAM are considerable but must be weighed against the best-possible and cost-effective strategies. Of great priority are strategies that integrate SAM management in community settings with additional child health and development interventions [13]. Given the close correlation and relationship between various forms of malnutrition (moderate and severe acute malnutrition), there is growing interest in common management protocols and simplified regimens for preventing and managing all forms of acute malnutrition. The sizeable subgroup of children with concurrent wasting and stunting represents a subgroup at much greater risk of adverse outcomes and mortality [14] and needs strategies that also integrate maternal and early child health and nutrition strategies.

There has been a healthy increase in research related to prevention and management strategies for SAM in recent years, all adding to the evidence base for effective implementation in field settings. Corresponding processes for guidelines development by WHO are understandably cautious, but it is worth noting that the guidelines for the management of SAM by WHO are now almost a decade old [15] and need updating as well as flexibility in implementation. Studies such as those by Hitchings and colleagues [1] should show the way to optimize the screening and management of SAM in settings with limited facilities and community capacity for weekly follow-up. The recognition that such infants may be at higher risk of relapse or mortality could well require additional contacts, such as fortnightly follow-up or outreach services, areas that should be studied in future evaluations.
